# On the Treatment of Placentia Prævia

**Published:** 1879-02

**Authors:** 


*On the Treatment of Placenta Prævia.* By Joseph T. Johnson, A. M., M. D., Professor of Obstetrics and Diseases of Women and Infants, Medical Dep't University of Georgetown ; Fellow of the American Gynæcological Society, etc.

This is a well written and interesting pamphlet on the above subject, read before the Medical Society of the District of Columbia. Premature induction of labor, and rupture of the amniolic sac, are points to which the author invites favorable consideration ; and he, by quotations and references, shows great familiarity with the literature of this most grave subject.

